# Immunological Barriers to Stem Cell Therapy in the Central Nervous System

**DOI:** 10.1155/2014/507905

**Published:** 2014-08-05

**Authors:** Gregory E. Tullis, Kathleen Spears, Mark D. Kirk

**Affiliations:** ^1^Division of Biological Sciences, University of Missouri, 102 LeFevre Hall, Columbia, MO 65211, USA; ^2^Department of Natural Sciences, Northwest Missouri State University, Maryville, MO 64468, USA

## Abstract

The central nervous system is vulnerable to many neurodegenerative disorders such as Alzheimer's disease that result in the extensive loss of neuronal cells. Stem cells have the ability to differentiate into many types of cells, which make them ideal for treating such disorders. Although stem cell therapy has shown some promising results in animal models for many brain disorders it has yet to translate into the clinic. A major hurdle to the translation of stem cell therapy into the clinic is the immune response faced by stem cell transplants. Here, we focus on immunological and related hurdles to stem cell therapies for central nervous system disorders.

Transplantation of cells, tissue, or organs between different individuals (allogeneic grafts) invariably leads to the rejection of the donor material due to a combination of humoral and cellular immune responses. In contrast, grafts from the same individual or an identical twin (autologous grafts) are rarely rejected. There are over 40 genes involved in graft rejection in humans. By far the most important are those encoding the major histocompatibility complex I and major histocompatibility complex II (MHC I and MHC II). In humans, MHC I and MHC II are also known as human leukocyte antigens (HLAs). MHC I and MHC II proteins are expressed on the surface of cells and contain small clefts in their extracellular domain that binds to small peptides. MHC I is comprised of one transmembrane, MHC protein, and one non-MHC protein called *β*2-microglobulin. MHC II is composed of two transmembrane, MHC proteins. MHC I molecules can only bind to peptides of 8 to 11 residues in length [1–3], whereas MHC II molecules have an open-ended groove that bind to larger peptides that are 10–30 residues long [4, 5]. However, 18–20 residues are the optimal peptide length for binding to MHC II [6]. Although they can only bind to one peptide at a time, both MHC I and MHC II can bind a variety of peptides in their clefts. MHC I proteins are expressed on almost all cells in the body including the central nervous system (CNS), where they are expressed on both glia and neurons* in vivo*. MHC I proteins are found on the surface of both axons and dendrites and are located in synapses both pre- and postsynaptically where they are involved in regulating neurite outgrowth [7]. MHC I proteins are also expressed on microglia following their activation by inflammatory stimuli, and they bind to peptides from the cytosolic proteosome in the endoplasmic reticulum (ER) where MHC I is synthesized (Figure 1). The MHC I-peptide complexes are transported to the cell membrane in exocytotic vesicles via the Golgi apparatus. The MHC I-peptide complexes can bind to T cell receptors (TCRs) on Cluster of Differentiation 8 (CD8) positive cytotoxic T cells and activate them. Unlike MHC I, MHC II proteins are only expressed in professional antigen presenting cells (APCs). These include dendritic cells, mononuclear phagocytes, B cells, endothelial cells, and thymic epithelial cells. Although MHC II proteins are expressed in the ER like MHC I, they do not usually bind to peptide fragments from the cytosol, because the peptide-binding sites in MHC II proteins are blocked by an inhibitor protein (Figure 2). MHC II proteins are transported through the Golgi complex and into exocytotic vesicles where they become exposed to peptides that derive from extracellular antigens taken up previously by endocytosis. The MHC II-peptide complexes are then expressed on the surface of the APCs where they can bind to TCRs on Cluster of Differentiation 4 (CD4) positive helper T cells.

MHC I and MHC II are highly variable in humans, because they both have multiple genes and each gene is highly polymorphic (Figure 3). MHC III genes are not involved in antigen presentation; however they are involved in the immune system. Some are components of the complement cascade, whereas others are cytokines and heat shock proteins. There are about 3500 alleles in MHC I and MHC II genes alone, which makes tissue matching (i.e., allomapping) very difficult between unrelated individuals [8, 9]. The MHC locus covers about 3500 kilobases on chromosome 6. There are three MHC I genes in humans (*HLA-A*,* HLA-B*, and* HLA-C*) that encode proteins that interact with T cells. There are also three MHC II loci (*HLA-DP*,* HLA-DQ*, and* HLA-DR*) that encode two proteins each (*α* and *β*). The binding of the TCRs on CD4^+^ helper T cells to the MHC II-peptide complex on APCs has different functions depending on the type of APC. Dendritic cells are the only APC that effectively activates naïve helper T cells. These activated T cells proliferate and differentiate into effector T cells, which bind to macrophages through the MHC II-peptide and activate them. Activated macrophages are larger and better able to phagocytose microbes [10]. Effector T cells are further activated to form memory T cells, which are long-lived cells that speed up the response to the same or similar peptide antigen. Effector T cells can also bind to the MHC II-peptide on B lymphocytes causing the B cells to produce more antibodies. B cells that display the same antigen on their B cell receptor and their MHC II proteins are further activated when an effector T cell binds to the MHC II-peptide. Note that although B cells can produce antibodies against a wide variety of antigens, MHC II proteins are restricted to peptides. The activated B cells differentiate into plasma cells, which produce large amounts of antibodies, and ultimately memory B cells [11, 12].

When cells from an allogeneic donor are transplanted into a host animal, many proteins including MHC I and MHC II will be seen as foreign by the host. These proteins will be processed in host dendritic cells and other APCs and presented on MHC I proteins to activate cytotoxic T cells. Other peptides will be presented on MHC II proteins to activate helper T cells. In addition to binding to self-MHC molecules containing foreign peptides, TCRs can bind directly to allogeneic MHC from the graft that contains either self or foreign peptides. The structure of allogeneic MHC-peptide complexes resembles self-MHC-foreign peptide complexes. Cells expressing MHC I and/or MHC II may be phagocytosed and processed by APCs in the host. MHC proteins are highly polymorphic and would be seen as foreign by host lymphocytes. Also, if the graft contains lymphocytes, a similar graft versus host response will be seen as well [13, 14].

Allogeneic grafts to the CNS are rejected similarly except that the adaptive immune response is delayed and less effective. The brain and eye are separated from blood by tight junctions between endothelial cells that line blood vessels. This forms the blood-brain barrier (BBB) and blood-retina barrier. A third barrier separates the cerebrospinal fluid from the blood (blood-cerebrospinal fluid barrier). Cerebrospinal fluid is secreted from the choroid plexus into the ventricles [15]. In contrast to the BBB where endothelial cells in the vessels are joined by tight junctions, the blood-cerebrospinal fluid barrier is formed by tight junctions between epithelial cells in the choroid plexus [16]. These barriers have a thick basement membrane and often astrocytes surround the capillaries in the CNS, which is referred to as the* glia limitans*. Small, hydrophobic molecules such as hormones can diffuse through the endothelial cell membranes into the CNS. The movement of many metabolic molecules like glucose across the endothelial cells is regulated by active transport. Small, aqueous molecules such as ions and small neuropeptides can diffuse through channels in the cell membrane or across tight junctions between cells. Larger proteins such as cytokines and antibodies cannot cross these barriers [17, 18]. Thus, antibodies cannot enter the CNS from the bloodstream. The CNS also has limited lymphatic drainage and dendritic cells, which are star-shaped cells that are constantly sampling their environment by pinocytosis. It was originally thought that the CNS was devoid of dendritic cells and perhaps that phagocytic microglia may serve a similar function in the CNS. However, it is now recognized that the CNS does have dendritic cells, but they are not distributed widely. They are primarily found in three types of areas in the brain: (1) areas of neurogenesis such as the subventricular zone, (2) areas that receive input from the environment such as olfactory nerve projections, and (3) areas of outside of the BBB [19]. They are also found in the juxtavascular parenchyma [20].

The CNS is not free from immune-surveillance for potential pathogens. For example, the frequency of CNS infections increases in immune-suppressed patients. Tissue transplanted into the brain, which would be tolerated by naïve animals, is rejected if the host was sensitized to alloantigens outside the CNS previously [21]. This suggests that T cells activated outside of the CNS find their way into the CNS and mount an immune response. Prior to this work, several authors showed that tissue transplanted in the brain did not provoke an immune response. This was called immune privilege or tolerance. It was thought that the CNS lacked an immune response, because it was walled off from the lymphatic and blood systems. Medawar was the first to show that immune responses can occur in the CNS. The humoral and cellular immune response in the CNS is delayed and less effective [22]. Thus, grafts to the CNS that Medawar and earlier researchers considered to be “tolerated” in their experiments (6–10 days) would have been rejected over longer time frames.

Encephalitogenic T helper cells that express interleukin (IL) 17 (Th17 cells) cross the blood-cerebrospinal fluid barrier in the choroid plexus as well as the BBB. Th17 cells are a distinct subset of helper T cells that play a key role in autoimmune disorders such as multiple sclerosis. Th17 cells also serve an important function at epithelial/mucosal barriers. They secrete cytokines that induce epithelial cells to secrete antimicrobial proteins. Most of the other T cells enter the CNS by crossing the BBB. Normally, lymphocytes are carried by the flow of blood in blood vessels. However, once CD4^+^ or CD8^+^ T lymphocytes are activated, they begin migrating along the walls of blood vessels. They initially attach by either tethering or rolling along the wall of blood vessels [23, 24]. This behavior is not observed in other parts of the body. The capture of T cells is initially mediated by the binding of *α*4-integrins on the T cell with vascular cell adhesion molecules and/or intracellular cell adhesion molecules 1 and 2 on the endothelial cell. Antibodies that disrupt this interaction lead to the detachment of the T cells. This initial binding must be quickly strengthened to withstand the shear forces associated with the flow of blood. This is done by the binding of cytokines on the luminal surface of the endothelial cells to cytokine receptors on the T cell. This ultimately leads to the expression of more integrins on the surface of the T cells. Once the T cells are captured, they begin crawling, usually against the flow of blood, and begin probing for a site to cross the endothelial cell layer (diapedesis). Blood flow is important for effective diapedesis. This crawling behavior is not unique to the CNS; however, the T cells tend to crawl further within the CNS before diapedesis, suggesting that the molecular cues for diapedesis are scarce in the healthy CNS. Diapedesis can occur either by T cells pushing their way through the tight junctions into the CNS (paracellular pathway) or by passing through a large pore in the endothelial cell (transcellular pathway), which is about 4-5 *μ*m. This transcellular pore is formed by the fusion of multiple, smaller pores that are induced by the crawling of T cells on the surface of the endothelial cells. This latter route appears to be the most common route through the BBB [23].

The adaptive immune system relies on its ability to distinguish between self- and non-self-antigens to initiate an immune response to eliminate potential pathogens in the body without damaging its own cells. An immune response that is too slow may lead to massive loss of cells due to an infection, whereas an immune response that is too fast and too aggressive may lead to massive loss of cells due to inflammation. Moderation of the immune response is the function of regulatory T cells (Tregs) [25]. In tissues like the dermis where new cells are constantly being produced, the loss of a few extra cells is not critical. However, in the CNS many neurons are terminally differentiated and not easily replaced; the loss of neural cells is debilitating if not fatal. Tregs have evolved to provide a counterbalance between the need for an immune response and the risk associated with it [26]. Tregs are T lymphocytes that express CD4^+^, the alpha chain of the interleukin-2 receptor (IL-2R*α*; a.k.a CD25), and the transcription factor forkhead box protein 3 (FoxP3). Ectopic expression of FoxP3 leads to the expression of proteins involved in suppressor activity such as cytotoxic T lymphocyte antigen 4 and glucocorticoid-induced tumor necrosis factor receptor-related protein. Tregs strongly inhibit the proliferation of responder T cells in coculture. Expression of surface markers, CD45RA and CD45RO, indicates naive and effector cells, respectively. CD45RO^+^ effector Tregs exhibit stronger suppressor activity than CD45RA^+^ naïve Tregs to TCR stimulation [27, 28]. The differentiation of effector/memory Tregs is dependent on antigen binding in the gut during the first 18 months of life [29]. Effector/memory Tregs can be further subdivided based on the expression of MHC II molecules (HLA-DR) on the cell surface. HLA-DR^−^ cells strongly suppress the proliferation of effector T cells, whereas HLA-DR^+^ induce T helper cells type 2 cells by secreting IL-4 and IL-10. Evidence of the crucial role of Tregs in immune suppression is that loss of FoxP3 activity results in a fatal, systemic autoimmune disorder called immune-dysregulation polyendocrinopathy X-linked syndrome. Activation of microglia attracts Tregs to the CNS through the production of C-C motif chemokine 22 that binds to C-C chemokine receptor 4 on Tregs [30, 31]. Also, astrocytes and neurons can induce Tregs to suppress autoreactive T cells in experimental autoimmune encephalomyelitis [32, 33].

Although wounds and autoimmune disorders are potentially amenable to stem cell therapy, in this review we have focused on cell transplants into healthy rodent brain tissue (Table 1), which is similar to transplanting stem cells into presymptomatic individuals years before the onset of neurodegeneration for many late-onset disorders like Alzheimer's and Parkinson's disease. The first attempts to treat brain disorders using tissue transplantation consisted of grafting neural tissue into the brain to treat either Parkinson's disease [34–37] or Alzheimer's disease [38, 39]. These allogeneic tissue grafts were rejected by a mixture of innate microglial cells and cytotoxic T cells (Table 1) [40, 41]. Host microglial cells infiltrate the necrotic tissue surrounding the grafts almost immediately after transplanting. Lymphocytes, dendritic cells, and host macrophages begin to accumulate around the graft soon (6–9 days after transplantation) after the blood vessels begin connecting to the host tissue. Many groups have used neural tissue from embryonic animals, because they appear not to express MHC I. However, these cells will express MHC I in the presence of proinflammatory cytokines. MHC I is expressed in these embryonic cells as early as 3 days after transplanting the tissue [41]. More recently, researchers have transplanted suspended cells instead of whole tissue grafts, because fewer cells die from necrosis. Encouragingly, blood vessels and neural connections form* de novo*. Allogeneic and xenogeneic cell grafts are usually rejected by an inflammatory, macrophage response instead of a cytotoxic T cell response as seen in tissue grafts (Table 1) [42–44].

Recently, our laboratory found that transplantation of embryonic stem cells (ESCs; 10^6^ cells) from CD-1 mice that have been neuralized by the addition of retinoic acid to the culture medium [45, 46] into the left striatum of two-month-old, allogeneic 129svj mice was rapidly rejected (7–14 days after transplantation) by the host immune system [47]. ESCs are the most primitive stem cells and can differentiate into most cell types found in the body. Undifferentiated ESCs can form teratomas following transplantation. Neuralized ESCs are a mixture of neural progenitor cells (NPCs) and differentiating neural cells [45]. Typically, neuralized ESCs of the B5 cell line (from the 129svj strain) do not form tumors following transplantation into the neural retina or CNS of allogeneic mice [45, 48]. In contrast, transplantation of 129svj neuralized ESCs into the striatum of syngeneic 129svj mice not only survived but proliferated over the course of 6 weeks* in vivo*. CD8^+^ cytotoxic T cells rapidly infiltrated allogeneic neuralized ESC grafts and these T cells remained well after the transplanted cells had been cleared. This is typical of a cellular immune response to grafts in other areas of the body [49]. Previously, it was thought that the innate immune system was responsible for rejecting cell grafts in the CNS. However, macrophages and microglia appeared to be absent from the allogeneic grafts. These results are reminiscent of earlier studies using neural tissue grafts rather than cell suspensions [41]. This may have to do with either the type of or number of cells used. We transplanted ten times more cells than in a related study [44]. Chen and colleagues transplanted NPCs, whereas Spears and colleagues transplanted ESCs that were neuralized by retinoic acid. Certainly, the type of cell transplanted can affect how and when they are rejected [48]. Rémy et al. [50] transplanted an equal number of aortic endothelial cells (from 30-day-old pigs), which express both MHC I and MHC II, and embryonic neurons (from e25–28 pigs) that expressed neither MHC I nor MHC II. The endothelial cells were rejected rapidly (3–7 days after transplantation) by primarily macrophages. In contrast, the neural cells were rejected more slowly (14–21 days after transplantation) by a combination of macrophages and cytotoxic T cells [50]. Undifferentiated ESCs express little or no MHC proteins; however, once they differentiate into neurons or other adult cells, they express the MHC I gene and are susceptible to killing by cytotoxic T cells [47, 51]. Many adult or somatic stem cells, like NPCs, also express MHC I and would be rejected similarly [52–55]. However, expression of MHC I alone is not sufficient to induce cell killing by cytotoxic T cells [56]. These results suggest that transplantation of allogeneic grafts into the CNS will require long-term immunosuppressive therapy as required for whole organ transplants in other parts of the body.

Results similar to those of Spears [47] were observed by Chen and coworkers [44]. They injected NPCs into the hippocampus of two-month-old BALB/c or C57BL/6 mice (10^5^ cells). NPCs express low levels of MHC I and MHC II. However, the amount of MHC I proteins increase in the presence of the proinflammatory cytokine interferon gamma (IFN*γ*). Chen et al. saw a 75% reduction in graft size after two weeks in allogeneic animals as compared to syngeneic. The authors refer to these as isogeneic grafts instead of syngeneic. Isogeneic generally refers to genetically identical sources such as identical twins. Inbred mice are highly similar, but not genetically identical. Thus, they will possess a high degree of sequence homology throughout the MHC locus. About 4% of the transplanted cells had differentiated into neurons, which express MHC I. However, unlike Spears [47], they also saw an increase in the number of activated microglia in allogeneic grafts as compared to syngeneic. Activated microglia secrete proinflammatory cytokines that inhibit neurogenesis in the dentate gyrus [57]. The mice were given nonsteroidal anti-inflammatory drugs (NSAIDs), indomethacin or rosiglitazone, orally once a day starting two days prior to the NPCs transplantation until the conclusion of the experiment (two weeks). Treatment with either NSAID greatly improved the survival of allogeneic grafts at two weeks. NSAIDs also increased the number of neurons in the grafts (NeuN^+^ cells) and neurogenesis in the hippocampus as measured by doublecortin expression. However, a commonly used immunosuppresive drug cyclosporine-A, which inhibits T cell activation (Figure 4), had little effect on survival of the allogeneic grafts and inhibited neurogenesis in the hippocampus. These results suggest that a longer course of NSAIDs would probably be necessary for treating humans following allogeneic stem cell grafts into the CNS. Immunosuppressive therapy renders patients vulnerable to tumor formation and infections. What effect would long-term use of NSAIDs have? Many NSAIDs can cause gastrointestinal tract ulceration and bleeding. NSAIDs are also associated with a relatively high incidence of renal problems. NSAIDs cause a constriction of the afferent arterioles and decreased renal perfusion pressure leading to high blood pressure, which increases the risk of stroke and heart failure.

Both cyclosporine-A and an anti-inflammatory corticosteroid, dexamethasone, either alone or in combination with each other, not only failed to prevent the activation of alloreactive, natural killer cells, but also strongly inhibited the differentiation of NPCs into mature neurons* in vitro* [58]. Most immunosuppressive drugs target either the TCR or IL-2 signaling pathways in T lymphocytes (Figure 4). Cyclosporine-A functions downstream of the TCR signaling pathway and inhibits the production of IL-2. Binding of MHC I or MHC II proteins to TCRs leads to an increase in IL-2 production. Binding of IL-2 to its receptor on the surface of T cells leads to the proliferation and differentiation of T cells. Cyclosporine-A is a small, fungal peptide that binds to a cellular protein called cyclophilin. The cyclophilin-cyclosporine-A complex binds to and inhibits the activity of calcineurin, a calcium-calmodulin-activated serine/threonine phosphatase. Calcineurin is required to activate the transcription factor, nuclear factor of activated T cells, which regulates the transcription of IL-2 and other cytokines. Although cyclosporine-A does reduce IL-2 synthesis, it is not very specific for this pathway. NPCs do not express either TCRs or the IL-2 receptor. Thus, inhibition of the differentiation of NPCs may be a side effect of cyclosporine-A. Immunosuppressive drugs that are more specific to the TCR and IL-2 pathways have been developed which might lack this side effect. These include mouse-human chimeric antibodies (a.k.a. humanized antibodies) that bind to the TCR and prevent it from binding to MHC I or MHC II. There are also humanized antibodies that bind to the IL-2 receptor and inhibit the receptor from binding to their ligand (IL-2). Similarly, a soluble fragment of the IL-2 receptor protein also binds to IL-2 and prevents IL-2 from binding of the IL-2 receptor. These humanized antibodies or soluble IL-2 receptor protein may not inhibit differentiation of NPCs like cyclosporine-A. Unfortunately, they are too big to cross the BBB into the CNS without modification [17]. However, drugs do not necessarily need to cross the BBB to have an effect in the CNS, if they induce effecter molecules that do cross the BBB. Additionally, many drugs that cross the BBB poorly are given at higher doses systemically to compensate. These higher dosages are likely to lead to further side effects of the drugs.

Whereas immunosuppressors like cyclosporine-A inhibit the adaptive immune system, both steroidal and nonsteroidal anti-inflammatory drugs inhibit the innate, nonadaptive immune system. Anti-inflammatory drugs inhibit the synthesis or secretion of proinflammatory cytokines, TNF*α* and IL-1, and their receptors as well as prostaglandins. Dexamethasone is a synthetic derivative of a small, hydrophobic hormone that is produced in the adrenal cortex of all vertebrates. NPCs do not express TNF*α* or IL-1 receptors, so how does dexamethasone inhibit differentiation of NPCs into neurons? Several new anti-inflammatory drugs are in development including soluble, cytokine receptors and humanized antibodies that bind to either TNF*α* or IL-1. These newer drugs will have to be tested for their ability to alter the differentiation of NPCs.

Although immune suppression prevents the rejection of allogeneic grafts, it also inhibits the normal restorative immune response in the CNS [59, 60]. For example, a patient is in an accident and suffers a minor laceration in the brain; the BBB is disrupted and plasma-derived thrombin, complement factors, as well as other components from the blood, flow into the neural tissue causing edema and an increase in intracranial pressure. Thrombin is normally expressed at low levels in the CNS and is involved in brain repair. However, at higher levels, it is toxic to neurons. Thrombin also binds to microglia and activates them [61]. These activated microglia secrete proinflammatory cytokines that attract macrophages and lymphocytes to the wound. Acute brain injury results in cell death both by necrosis and by apoptosis. If these apoptotic cells are not quickly cleared by microglia and macrophages, then they will release more neurotoxins and apoptotic signals. Activated microglia also activate astrocytes, which begin walling off the wound (gliosis). Endothelial cells begin reforming tight junctions and reestablishing the BBB. Administration of anti-inflammatory drugs will greatly reduce pain due to swelling and an increase in intracranial pressure. Likewise, if the patient is on immunosuppressive therapy, it will result in a delay in the activation of cytotoxic T cells that induce apoptosis in defective cells.

Although many studies report detrimental effects of macrophages and microglia on repair from neurotrauma and chronic neurodegeneration, primarily due to the proinflammatory cytokines that they secrete, there are also many studies that report a positive influence by these cells on recovery from CNS trauma. For instance, the proinflammatory cytokines, TNF*α* and IFN*γ*, secreted by these cells not only attract immune cells to the site of injury but also attract NPCs [62]. Additionally, other cytokines such as monocyte chemoattractant protein-1 can also act on NPCs [63]. Loss of the ability of the stem cells to home to the site of injury greatly impairs their effectiveness. The simplest solution to this problem is to use autologous stem cells, which would not elicit an immune response. NPCs can differentiate into all cell types found in the brain, but NPCs are found primarily in three rather inaccessible regions in the CNS: the dentate gyrus in the hippocampus, the subventricular zone, and the pericentral canal in the spinal cord. Alternately, mesenchymal stem cells (MSCs) are of particular interest, because they appear to* trans*-differentiate into neurons both* in vitro* and* in vivo *[64], although there is some doubt about this kind of reprogramming* in vivo* [65]. Undifferentiated MSCs can also express markers characteristic of neural differentiation [66]. Note that Coyne et al. showed that traditional cell labels, 5-bromo-2-deoxyuridine and bis-benzamide, are passed from lysed MSCs to host phagocytes, astrocytes, and even neurons in the absence of viable cells [42].

MSCs have beneficial trophic effects in the CNS regardless of whether they can replace lost neurons. This is presumably due to the cytokines they secrete. MSCs secrete brain-derived neurotrophic factor and beta nerve growth factor [67]. Another attractive aspect of using MSCs is that they are relatively easy to isolate from bone marrow, adipose tissue, umbilical cord blood, and dermis. Most of the early work on MSCs focused on MSCs from bone marrow. However, recently several articles have focused on adipose tissue, because of the ease of harvesting this tissue. Adipose MSCs have a plasticity similar to that of bone marrow-derived MSCs [68]. Obviously, autologous ESCs are not a viable source because they would have to be isolated from embryonic tissue ahead of time. However, a great deal of excitement in this field has focused on induced pluripotent cells (iPSCs) that can be generated from adult somatic cells by the expression of four transcription factors [69, 70]. Unfortunately, two of these transcription factors are oncogenic. However, small molecule based methods to generate iPSCs are being developed, and although these iPSCs are not ready for use in humans due to the potential for tumor formation, this technology is advancing rapidly [71]. In fact, the first clinical trial of iPSCs to treat age-related macular degeneration was recently approved in Japan [72].

Autologous NPCs or MSCs transplants require a biopsy to harvest the stem cells, propagating the cells* in vitro*, and a second surgery to inject the cells in the problem area. If the stem cells could be altered such that the same cells could be used in multiple recipients, then the cells could be propagated on a much larger scale. Much success has occurred using allogeneic or xenogeneic grafts from embryonic neural tissue for treating Parkinson's disease [73]. Functional recovery was dependent on the embryonic age of the donor tissue (e26-27 for pigs) [74]. This is primarily due to the fact that embryonic tissue expresses little or no MHC I and MHC II, as mentioned above. However, MHC I and MHC II expression in these cells can be upregulated by proinflammatory cytokines, IFN*γ* and TNF*α*, as would occur at the site of implantation [75, 76]. One solution to this problem would be to eliminate MHC I and MHC II expression from these cells entirely. However, these cells and their progeny would be resistant to killing by the host immune system, if they became tumorigenic or infected. Another potential solution would be to transplant Tregs together with allogeneic or xenogeneic stem cells. CD4^+^ and FoxP3^+^Tregs are divided into 2 subgroups: naturally occurring Tregs that are selected in the thymus like other effector T cells and induced Tregs that are generated outside the thymus from CD4^+^ and FoxP3^−^ Tregs. Induced Tregs are converted by TGF-*β*, IL-2, retinoic acid, or leukemia inhibitory factor to tolerate their antigen rather than reject it [77]. Unfortunately, there are only a small number of naturally occurring Tregs in the body and they are difficult to isolate. A better approach would be to isolate CD4^+^ T cells from the donor or patient and induce them to become Tregs by the addition of cytokines or retinoic acid before transplanting the cells. The question is how stable will these Tregs be after transplantation? For instance, it may be necessary to reprogram these cells by expressing FoxP3 from a constitutive promoter to maintain their Treg phenotype* in vivo*.

In summary, organ transplants have been quite successful, if the donor and recipient are sufficiently matched in the MHC region and immune suppression is applied. Allogeneic and xenogeneic tissues transplanted in the CNS are eventually rejected in the absence of immune suppression, even though the immune response in the CNS is modest relative to the remainder of the body. The challenges facing intracerebral transplantation are multifocal. Measures must also be taken to limit secondary damage in the brain from tissue edema and inflammation. This requires anti-inflammatory as well as immunosuppressive therapy. Another problem in treating CNS disorders with brain grafts is finding effective therapeutics that can regulate secondary complications due to the insult to the host tissue and the presence of foreign cells that can also cross the BBB. Small molecules like NSAIDs and peptides can diffuse across the BBB, but this is not very efficient. Larger molecules like humanized antibodies cannot cross the BBB without modification. Further research is necessary before brain grafts can be used to treat CNS disorders effectively.

## Figures and Tables

**Figure 1:**
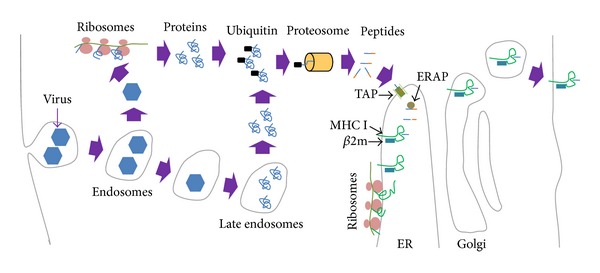
MHC I antigen presentation pathway. In this diagram the peptides derive from viruses that are taken into the cell by endocytosis. However, this could be any protein. The virus escapes the endosomes into the cytoplasm where it is transcribed and translated (ribosomes at the top). Some proteins also escape later into the cytoplasm. As these proteins become dysfunctional, they are labeled with ubiquitin (black rectangle) and transported to the proteosome in which the proteins are degraded into small peptides. These peptides translocated into the endoplasmic reticulum (ER) by the transporter associated with antigen processing (TAP). They are cleaved further in the ER by the ER associated peptidase (ERAP). MHC I is synthesized in the ER and dimerizes with beta-2-microglobulin (*β*2m). MHC I binds to small peptides in the ER and the MHC I-peptide complexes are transported to the cell surface in exocytic vesicles.

**Figure 2:**
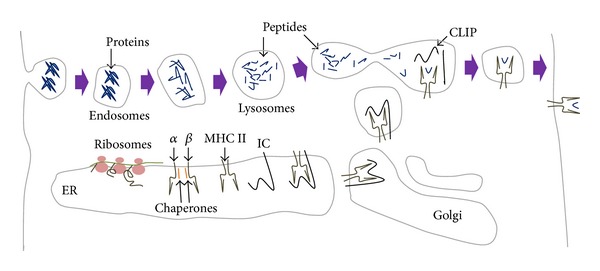
MHC II antigen presentation pathway. MHC II is synthesized in the endoplasmic reticulum (ER) as a heterodimer (*α*, *β*). MHC II proteins are also bound to the invariant chain protein (IC), which inhibits the binding of peptides to the MHC II proteins. IC is cleaved after the exocytic vesicles fuse with the acidic lysosomes to form class II invariant chain protein (CLIP). These MHC II proteins bind to small peptides (10–30 a.a.) in the vesicle and appear on the cell surface when these vesicles fuse with the plasma membrane.

**Figure 3:**
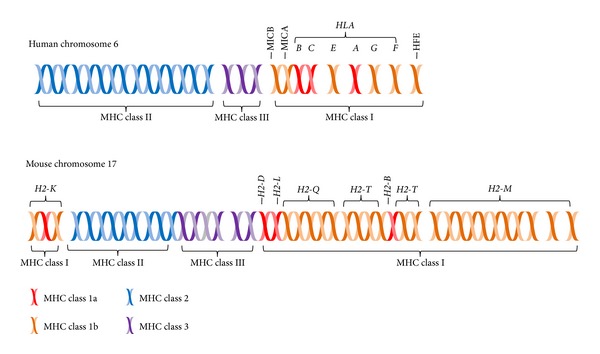
Comparison of MHC regions on chromosome 6 in humans and chromosome 17 in mice. The classical MHC class Ia genes (red), which include* HLA-A*,* HLA-B*, and* HLA-C* in humans and* H2-K*,* H2-D*,* H2-L*, and* H2-B* in mice, are much more polymorphic than the nonclassical, MHC class Ib genes (orange).* H2-L* is closely related to* H2-D* and is only found in BALB/c mice. MHC class III proteins are not involved in antigen presentation. This figure was adapted from Elmer and McAllister [7].

**Figure 4:**
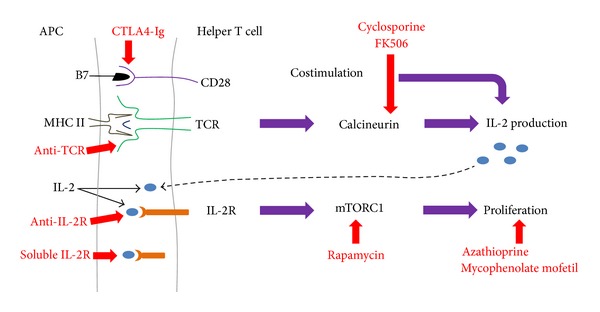
Points of action of immunosuppressive drugs. Various types of immunosuppressive drugs are shown in red and their targets are indicated by red arrows. In this example, we are showing the interaction of T cell receptor (TCR) proteins with MHC II-peptide complexes on the surface of antigen presenting cells (APC). TCRs also bind to MHC I-peptides on CD8^+^ T cells. The TCR signal is enhanced by the binding of B7 on the surface of APCs to CD28 on T cells. The binding of IL-2 to its receptor (IL-2R) on T cells results in proliferation and differentiation of the T cells into effector and memory cells. Humans express a second form of IL-2R that is soluble in serum, because it is missing the transmembrane domain of Il-2R. Soluble IL-2R binds and reduces the serum levels of IL-2.

**(a):** 

Source	Year	Transplants			Recipients		
		Species	Strain	Tissue used	Species	Strain	Location
Nicholas et al. [40]	1987	Mice	BALB/cANC	Embryonic neocortex These cells do not express MHC I.	Mice	BALB/cANC 6–8-week-old female mice	Right lateral ventricle
			CBA/J				
			E15–18				
		*Results *					
		Significantly more inflammatory cells in allogeneic than in syngeneic grafts. There were some T cells in the allogeneic grafts, but not syngeneic grafts. More neurons survive in syngeneic versus allogeneic grafts. There is less necrosis as well.					

Lawrence et al. [41]	1990	Rat	PVG	Embryonic hippocamus	rat	AO	Hippocampus
			AO	(E18–20)-no MHC I			
		*Results *					
		Allografts were rejected by both macrophages and cytotoxic T cells. Grafts were infiltrated early (3–6 d) by host microglia especially in necrotic tissue surrounding the graft. Blood vessels begin to join the graft to the host as early as 1 day. Lymphocytes, macrophages, and microglia begin to accumulate around the larger vessels. MHC I expression is turned on 3–6 days after transplanting. Most cells in the allografts were rejected by cytotoxic T cells by 27 d.					

**(b):** 

Source	Year	Transplants		Recipients			Number of cells
		Species	Cell type	Species	Strain	Location	
Rémy et al. [50]	2001	Pig	Aortic endothelial cells from 30 d old pigs express both MHC I and MHC II	Rat	LEW.1A	Striatum	400,000
		Pig	Embryonic neurons from ventral mesencephalon e25–28 express little or no MHC I.				400,000
		*Results *					
		Endothelial cells were rejected quickly (3–7 days) by primarily macrophage/microglia cells. Neurons were rejected much later (14–21 days) by both macrophages and cytotoxic lymphocytes.					

**(c):** 

Source	Year	Transplants			Recipients			Number of cells
		Species	Strain	Cell type	Species	Strain	Location	
Coyne et al. [42]	2006	Rat	Sprague-	Bone marrow MSCs from eGFP rats.	Rat	Sprague-Dawley	Striatum or hippocampus	50,000
			Dawley					
		Sprague-Dawley rats are outbred instead of inbred so these are allogeneic grafts and not syngeneic (225 g females).						
		*Results *						
		Few GFP-positive MSCs were seen at either location at 7 days and almost none at 14 days after transplanting. MSCs elicited an inflammatory response. Activated macrophages and microglia were seen in the grafts.						

Tambuyzer et al. [43]	2009	Mice	C57BL/6	Marrow stromal cells	Mice	FVB	Intracerebral right pelvic limb muscle	200,000
		*Results *						
		Stromal cells are lost over 3-4 weeks. Activation of microglia cells (CD11b). Muscle grafts were rejected by T cells, but not cerebral grafts.						

Chen et al. [44]	2011	Mice	C57BL/6	Neural progenitor cells that express eGFP from CAG promoter	Mice	C57BL/6	Hippocampus	100,000
						Balb/c		
		*Results *						
		Fewer GFP-positive cells in allogeneic grafts as compared to syngeneic. More activated microglia in allografts than syngeneic one. Neurogenesis is inhibited in allografts. This effect is reversed with NSAIDs.						

Spears [47]	2011	Mice	129svj	Neuralized ESCs	Mice	129svj	Left striatum	1,000,000
				expressing eGFP.		CD-1		
		*Results *						
		Allografts were rejected quickly (5–7 days) by cytotoxic T cells. Little activated microglia were detected. Syngeneic grafts increase in size.						
